# In Silico Analysis of Tumor Necrosis Factor α-Induced Protein 8-Like-1 (TIPE1) Protein

**DOI:** 10.1371/journal.pone.0134114

**Published:** 2015-07-24

**Authors:** Pei Shen, Hong Zhang, Zhaoliang Su, Shengjun Wang, Huaxi Xu

**Affiliations:** 1 Department of Immunology, Institute of Laboratory Medicine, Jiangsu University, Zhenjiang, Jiangsu, People’s Republic of China; 2 Department of Clinical Laboratory, Nantong Rich Hospital, The Fourth Clinical College of Yangzhou University, Nantong, Jiangsu, People’s Republic of China; 3 Laboratory Medicine Center, Affiliated Hospital of Nantong University, Nantong, Jiangsu, People’s Republic of China; Russian Academy of Sciences, Institute for Biological Instrumentation, RUSSIAN FEDERATION

## Abstract

Tumor necrosis factor α-induced protein 8 (TNFAIP8)-like protein 1 (TIPE1) was a member of TNFAIP8 family. Previous studies have shown that TIPE1 could induce apoptosis in hepatocellular carcinoma. In this study, we attempted to predict its potential structure. Bioinformatic analysis of TIPE1 was performed to predict its potential structure using the bioinfomatic web services or softwares. The results showed that the amino acid sequences of TIPE1 were well conserved in mammals. No signal peptide and no transmembrane domain existed in human TIPE1. The aliphatic index of TIPE1 was 100.75 and the theoretical pI was 9.57. TIPE1 was a kind of stable protein and its grand average of hydropathicity was -0.108. Various post-translational modifications were also speculated to exist in TIPE1. In addition, the results of Swiss-Model Server and Swiss-Pdb Viewer program revealed that the predicted three-dimensional structure of TIPE1 protein was stable and it may accord with the rule of stereochemistry. TIPE1 was predicted to interact with FBXW5, caspase8 and so on. In conclusion, TIPE1 may be a stable protein with no signal peptide and no transmembrane domain. The bioinformatic analysis of TIPE1 will provide the basis for the further study on the function of TIPE1.

## Introduction

Tumor necrosis factor α-induced protein 8 (TNFAIP8), the first member of TNFAIP8 family, was originally identified by comparing two primary and two matched metastatic head and neck squamous cell carcinoma (HNSCC) cell lines using the methods of northern blot and differential display of mRNAs [1]. Then the other members of TNFAIP8 family were identified successively by the method of high throughput gene microarray technology [2]. To date, the family of TNFAIP8 contains at least four members, including TNFAIP8 (also called SCC-S2), TNFAIP8-like protein 1 (TNFAIP8 l1, TIPE1), TNFAIP8-like protein 2 (TNFAIP8 l2, TIPE2), TNFAIP8-like protein 3 (TNFAIP8 l3, TIPE3) [2–5]. All the members of TNFAIP8 family share homologous sequence at the high degrees and they are involved in multiple cell behaviors, especially cell apoptosis and cell proliferation [2,3,6].

Previous studies have focused on the functional and structural studies of TNFAIP8 and TIPE2. Kumar et al. have demonstrated that TNFAIP8 contains a death effector domain (DED) and it is a negative regulator of apoptosis in certain cell types [3]. Sun et al. have reported that TIPE2 is a negative regulator to be involved in the regulation of immune homeostasis [2]. The function of TIPE2 in immunity may be associated with its hydrophobic central cavity, which could bind to a cofactor [7]. Recently, Fayngerts et al. also showed that TIPE3 functioned as a transfer protein for phosphoinositide second messengers with a larger hydrophobic cavity and its expression was increased in human cancer cells [8]. Although previous studies have shown that TIPE1 could induce apoptosis in hepatocellular carcinoma (HCC) cells by regulating Rac1, little is known about its structure [9]. Hence, we attempted to predict its potential structure by using the method of bioinfomatics in this study.

## Materials and Methods

### Sequences retrieval and homology tree construction

All the sequences of TIPE1 from various species, including Human, Bos taurus, Papio anubis, Rattus norvegicus, Pan troglodytes, Mustela putorius, Macaca fascicularis, Monodelphis domestica, Ficedula albicollis, Ovis aries, Felis catus, Mus musculus, Cricetulus griseus and Macaca mulatta, were obtained from NCBI. Multiple sequence alignments were performed using DNAMAN software and homology tree was constructed.

### Bioinformatics analysis of TIPE1

The signal peptide of human TIPE1 was predicted by SignaIP-4.1 Server of Center for Biological Sequence Analysis (CBS) platform and the web site of the tool is http://www.cbs.dtu.dk/services/SignalP/. TMpred program (http://www.ch.embnet.org/software/TMPRED_form.html) was then used to predict the transmembrane region and its associated orientation of TIPE1. The hydrophilicity, accessibility, polarity, flexibility, mutability, bulkiness and refractivity of TIPE1 were predicted using Protscale Server in expasy platform (http://web.expasy.org/protscale/). Post-translational modifications of TIPE1 protein, including mannosylation sites, glycosylation sites, phosphorylation sites and kinase specific phosphorylation sites, etc, were also predicted in CBS predicted Server (http://www.cbs.dtu.dk/services/). Then various physiological parameters of TIPE1 were validated by Protparam Server from expasy platform (http://web.expasy.org/protparam/).

### Homology modeling and analysis of TIPE1

Homology modeling of TIPE1 was performed using Swiss-Model of expasy platform (http://swissmodel.expasy.org/). Then the predicted result was analyzed by Ramachandran plot of Swiss-PdbViewer program.

### Protein-protein interaction analysis for TIPE1

Protein-protein interaction study was performed using Search Tool for Retrieval of Interacting Genes and proteins (STRING), which is a database of known and predicted protein interactions.

## Results

### Sequences retrieval and homology tree construction

Of all the sequences, the amino sequence of human TIPE1 (NP_689575 and NP_001161414) is provided as follows: MDTFSTKSLALQAQKKLLSKMASKAVVAVLVDDTSSEVLDELYRATREFTRSRKEAQKMLKNLVKVALKLGLLLRGDQLGGEELALLRRFRHRARCLAMTAVSFHQVDFTFDRRVLAAGLLECRDLLHQAVGPHLTAKSHGRINHVFGHLADCDFLAALYGPAEPYRSHLRRICEGLGRMLDEGSL-

**Fig 1:**
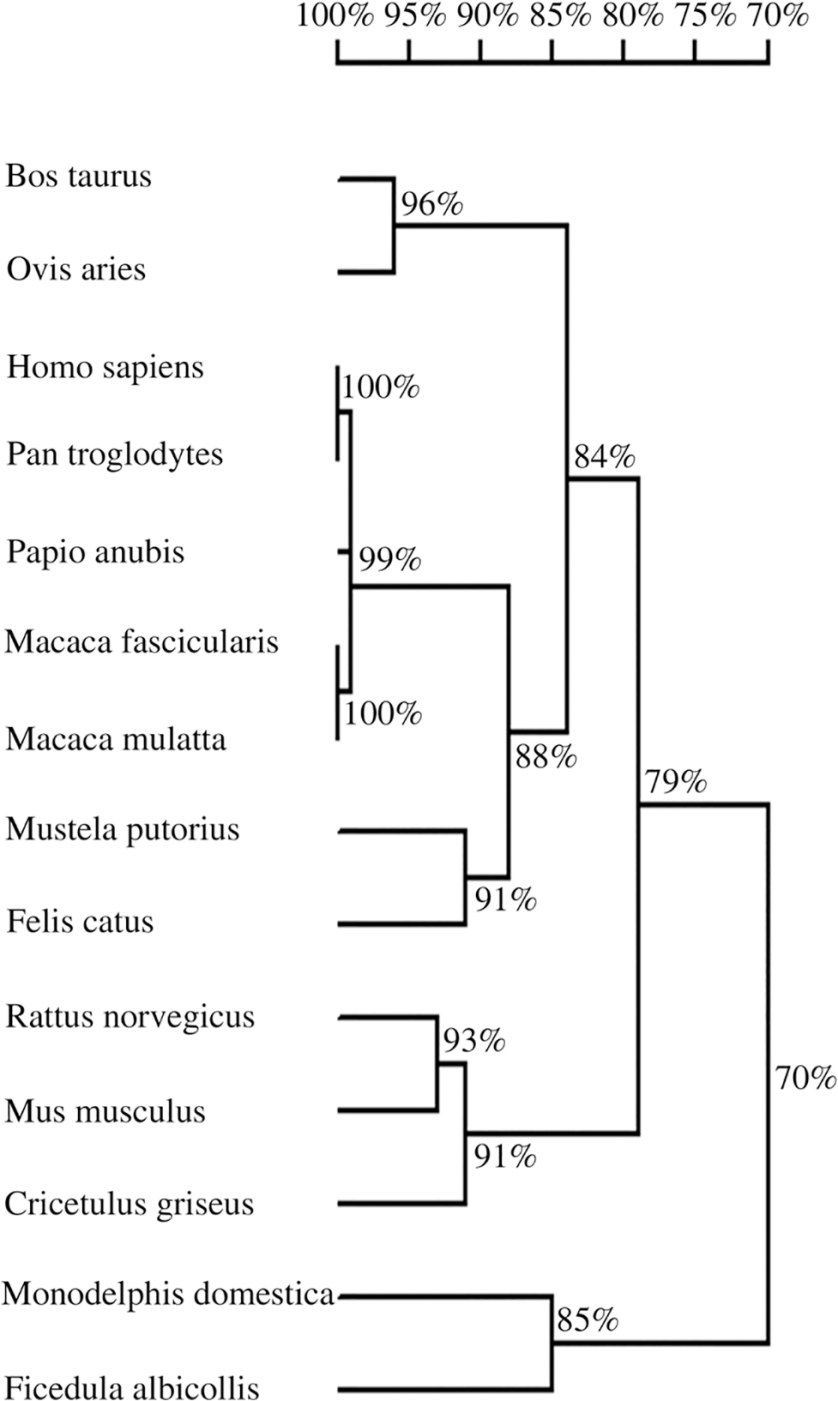
Homology tree construction. Homology tree is constructed by DNAMAN software and the homology of TIPE1 from various species is shown.

The sequences from various species were aligned using DNAMAN software and then homology tree was constructed using DNAMAN to show the homology of TIPE1 from various species. As the results shown in Fig 1, TIPE1 from various species share a high degree of sequence homology and its amino acid sequence is well conserved in mammals.

### Prediction of signal peptide and transmembrane domain of TIPE1

**Fig 2:**
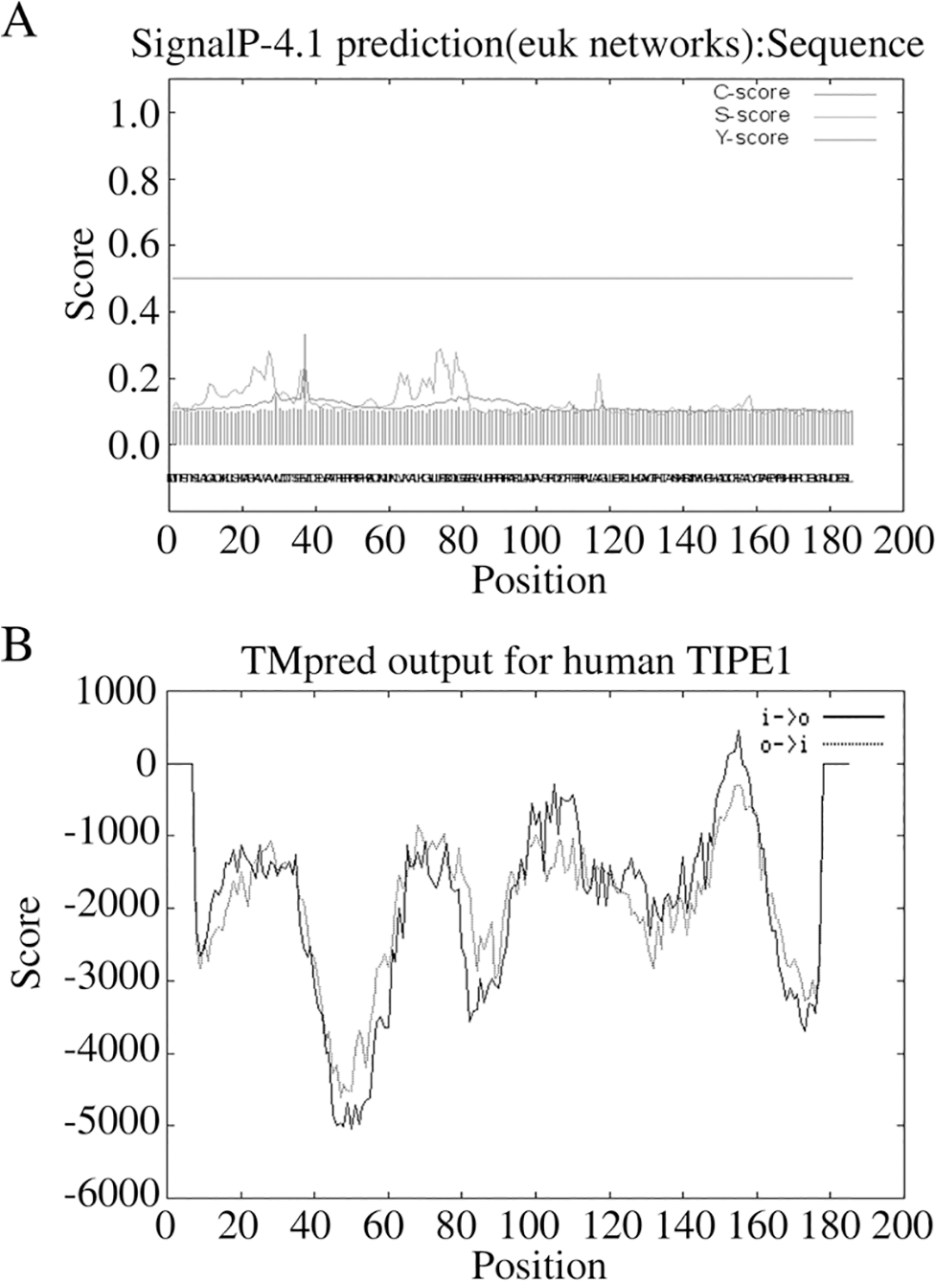
Prediction of signal peptide and transmembrane domain of TIPE1. Signal peptide (A) and transmembrane domain (B) of TIPE1 are predicted by SignaIP Server and TMpred Server, respectively. X axis represents amino acid sequence from N- to C- terminal. Y axis represents scores computed by each server.

The signal peptide of TIPE1 was predicted using SignaIP Server [10] and the results were shown as Fig 2. All the scores obtained from SignaIP Server, including C-, S- and Y- score were all less than the standard value (0.5). And the results of Fig 2A indicated that no signal peptide existed in TIPE1 sequence.

Then the transmembrane domain of TIPE1 was predicted using TMpred Server. As the results shown in Fig 2B, the max value was 459 and the associated core region was from 146 aa to 166 aa position. It may be a possible transmembarne helix from inside to outside. However, only the prediction scores above 500 are considered to be significant in TMpred server, and the helix from 146 aa to 166 aa may be insignificant, since its value was 459.

### Prediction of hydrophilicity, accessibility, polarity, flexibility, mutability and bulkiness of TIPE1

**Fig 3:**
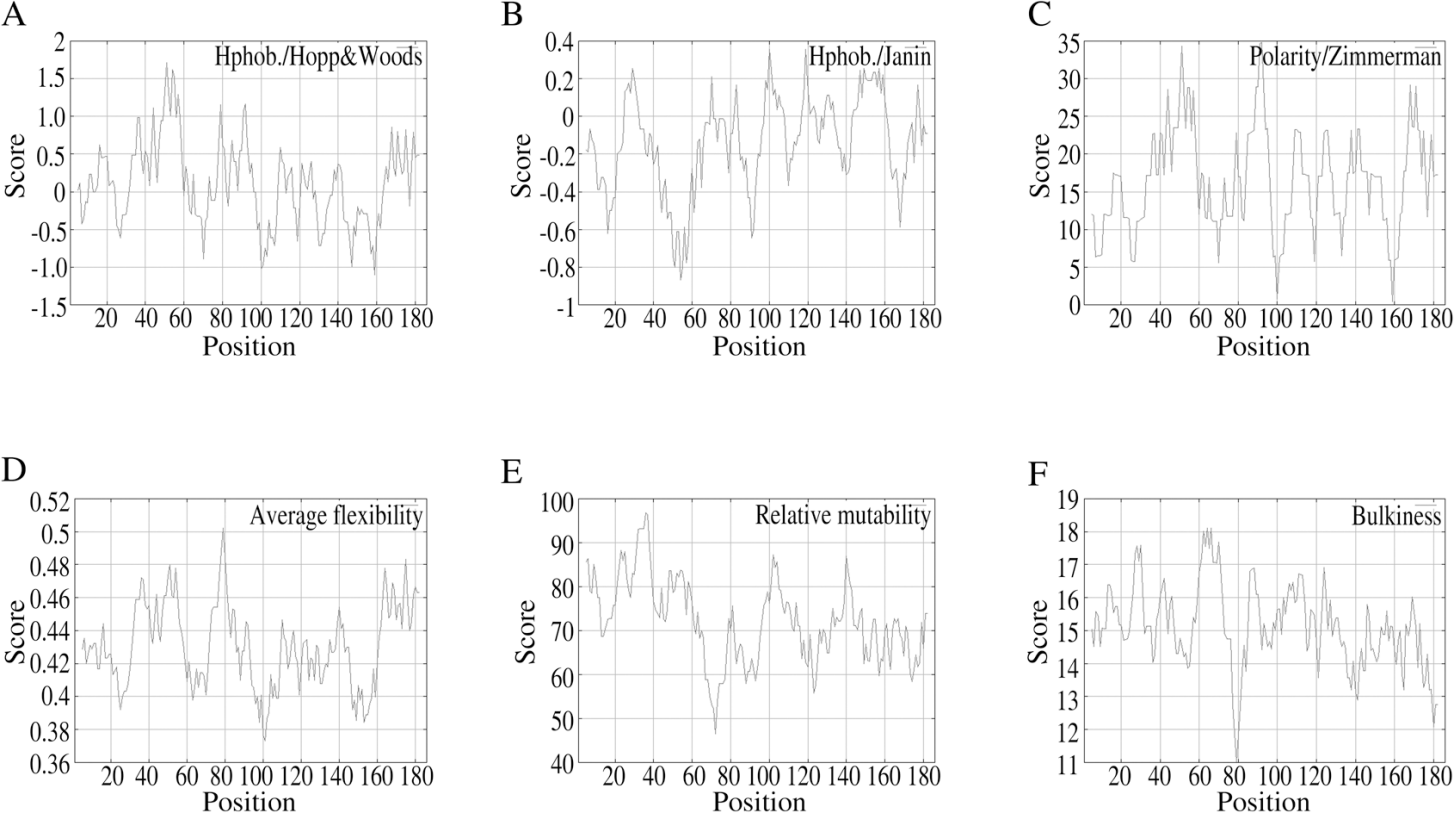
Prediction of hydrophilicity, accessibility, polarity, flexibility, mutability and bulkiness of TIPE1. The hydrophilicity (A), accessibility (B), polarity (C), flexibility (D), mutability (E) and bulkiness (F) of TIPE1 are predicted using Protscale Server of expasy platform. X axis represents amino acid sequence from N- to C- terminal. Y axis represents scores computed by each algorithm.

Protscale Server provided by expasy platform was then used to analyze the hydrophilicity, accessibility, polarity, flexibility, mutability and bulkiness of TIPE1. The higher score means the higher probability of each parameter of TIPE1. As the results, the hydrophilicity values (Fig 3A) are between -1.100 (position 159 aa) and 1.711 (position 51 aa). The accessibility value (Fig 3B) obtained by Janin score represents the free energy of transfer from inside to outside of a globular protein and the values are between -0.867 (position 54 aa) and 0.356 (position 100 aa). The polarity values (Fig 3C) are between 0.422 (position 159 aa) and 34.826 (position 92 aa), while the average flexibility values (Fig 3D) are between 0.373 (position 101 aa) and 0.502 (position 79 aa). The relative mutability values (Fig 3E) are between 46.556 (position 72 aa) and 96.778 (position 36 aa). The bulkiness values (Fig 3F) are between 11.017 (position 79 aa) and 18.120 (position 64 and 66 aa).

### Post-translational modifications of TIPE1

Various post-translational modifications were then predicted in CBS Prediction Server. Firstly, N-terminal acetylation site was predicted using NetAcet Server and the results showed that no acetylation site existed in TIPE1. Secondly, C-mannosylation site was predicted using NetCGlyc Server and N-linked glycosylation site of TIPE1was predicted using NetNGlyc Server. However, neither C-mannosylation site nor N-linked glycosylation site could be found in the amino acid sequence of TIPE1. In parallel, NetOGlyc Server was used to predict the mucin type O-GalNAc glycosylation site in TIPE1 and the results demonstrated that three O-GalNAc glycosylation sites existed in TIPE1 at the positions of 42 aa, 46 aa, 54 aa and 268 aa.

**Fig 4:**
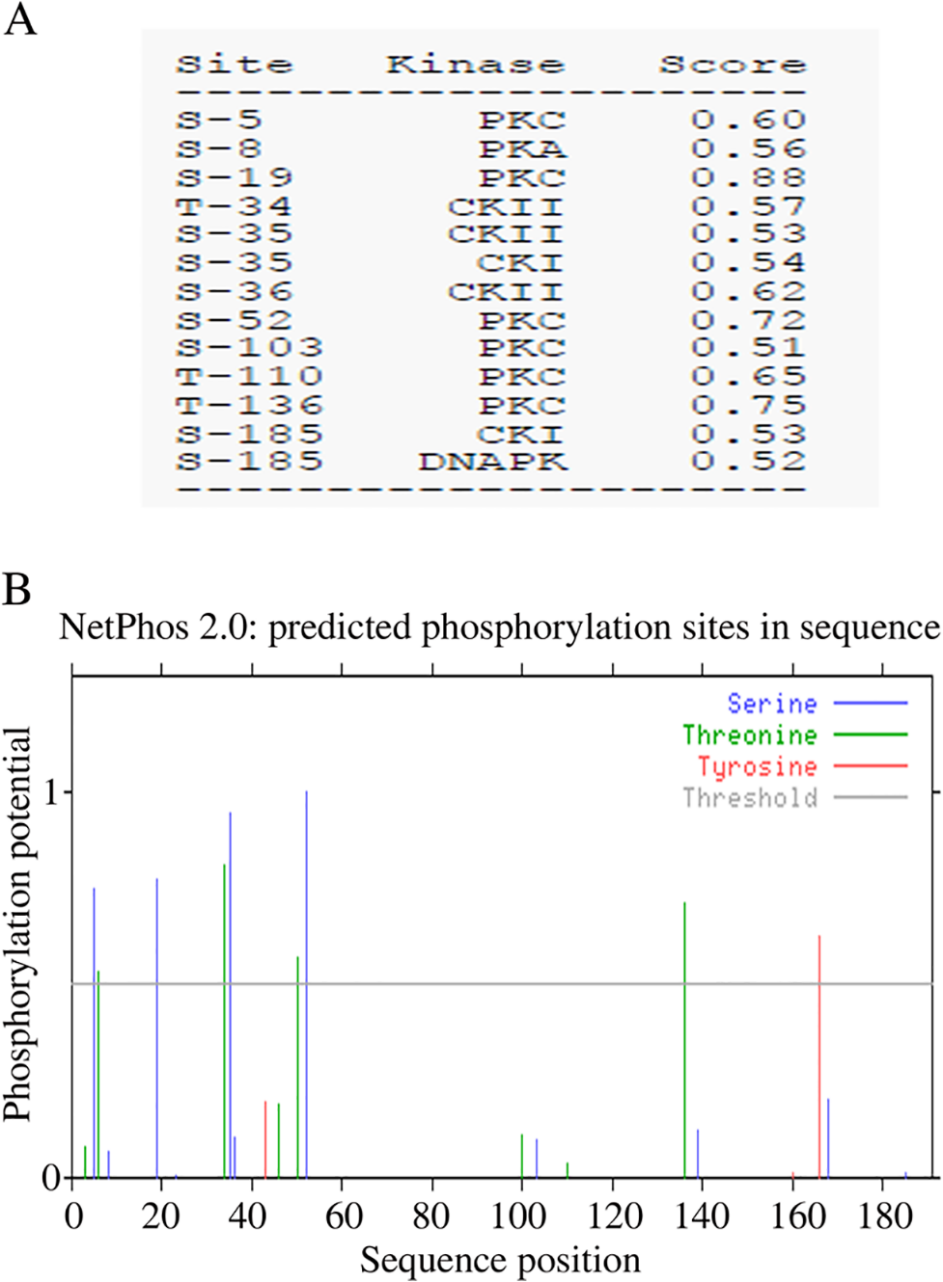
Post-translational modifications of TIPE1. (A) NetPhosK Server was performed to predict kinase specific phosphorylation sites in TIPE1. (B) NetPhos Server was performed to predict generic phosphorylation sites in TIPE1. X axis represents amino acid sequence from N- to C- terminal. Y axis represents values computed by the software. The values above the threshold mean the most potential of phosphorylation.

In addition, NetPhos Server and NetPhosK Server were also conducted to analyze the generic phosphorylation sites and kinase specific phosphorylation sites in TIPE1. As the results, protein kinase C (PKC) sites (position 5 aa, 19 aa, 52 aa, 103 aa, 110 aa, 136 aa) and Protein kinase A (PKA) site (position 8 aa) were also predicted to be existed in TIPE1 protein (Fig 4A). However, no p38 MAPK and Protein kinase B (PKB) site could be found in TIPE1. Meanwhile, four Serine phosphorylation sites (position 5 aa, 19 aa, 35 aa and 52 aa), four Threonine phosphorylation sites (position 6 aa, 34 aa, 50 aa and 136 aa) and one Tyrosine phosphorylation site (position 166 aa) might exist in TIPE1 (Fig 4B).

### Physiological parameters of TIPE1

ProtParam Server is used to predict various physiological parameters. As the results, the predicted molecular weight is 20827.2 dalton and the theoretical pI is 9.57. The speculated formula is C_918_H_1505_N_275_O_259_S_9_ and the total number of atoms is 2966. The estimated half life of TIPE1 is 30 hours (mammalian reticulocytes, in vitro) and > 20 hours (yeast, in vivo), respectively. In addition, the instability index is predicted to be 35.94 and this indicates that TIPE1 is a stable protein. Meanwhile, the aliphatic index is 100.75, while the grand average of hydropathicity (GRAVY) is -0.108.

### Homology modeling and evaluation of model stability

**Fig 5:**
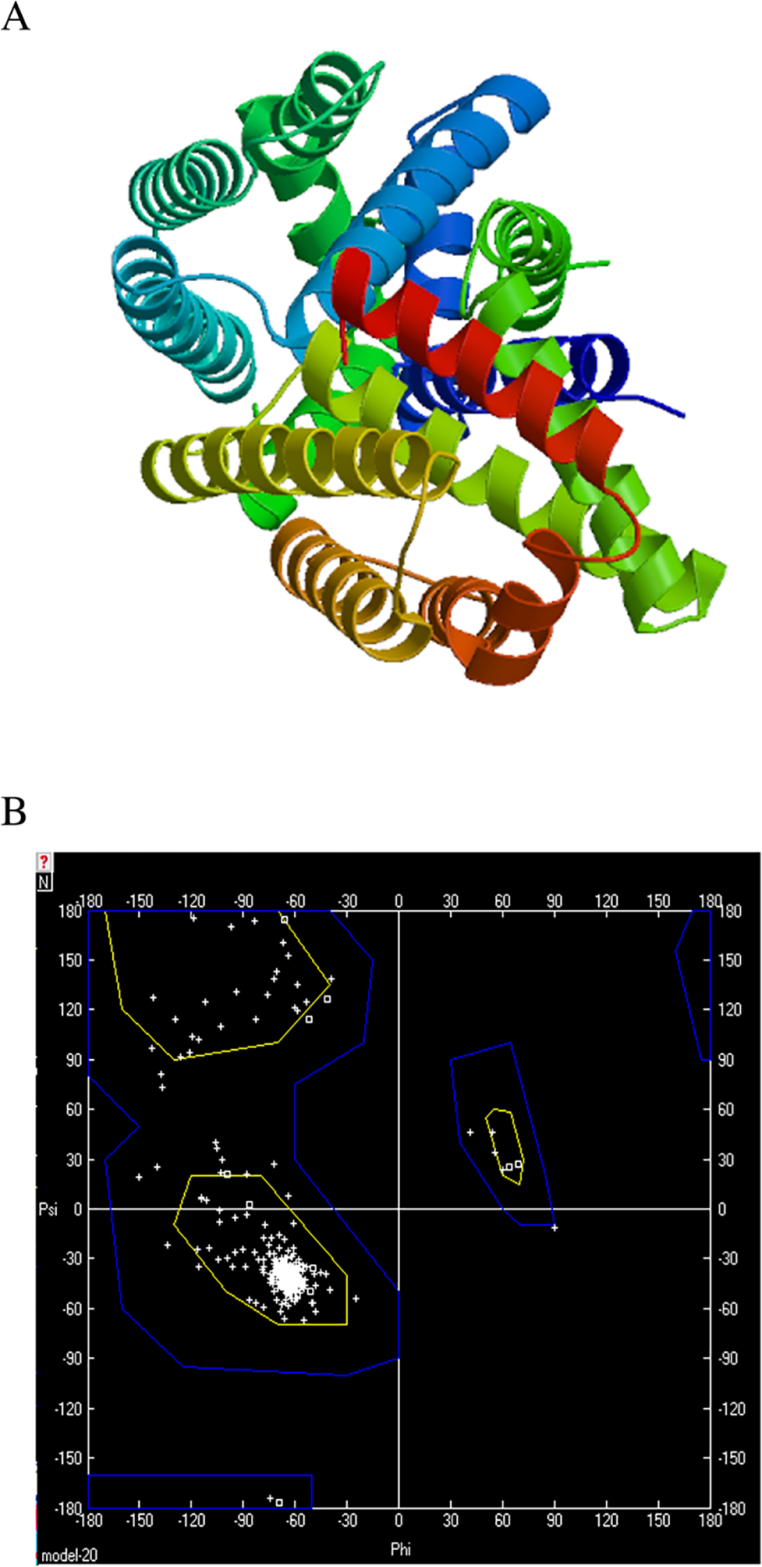
Homology modeling and evaluation of model stability. (A) Homology modeling was performed by Swiss-Model Server and the predicted 3D structure of TIPE1 protein was shown. (B) Model quality was evaluated using the method of Ramachandran plot and the results represent the acceptable stability of 3D structure of TIPE1 protein.

Homology modeling was performed by Swiss-Model Server in expasy web and the predicted three-dimensional (3D) structure of TIPE1 protein was shown in Fig 5A. Then the model quality was evaluated using the method of Ramachandran plot (Fig 5B) and the results showed that TIPE1 had 0.6% residues in outlier region. The value obtained by the method of energy minimization was -20599.754 KJ/mol. Based on these data, the results of Ramachandran plot revealed that the modeled 3D structure of TIPE1 protein might have the acceptable stability and it conforms to the rule of stereochemistry.

### Protein-protein interaction analysis for TIPE1

**Fig 6:**
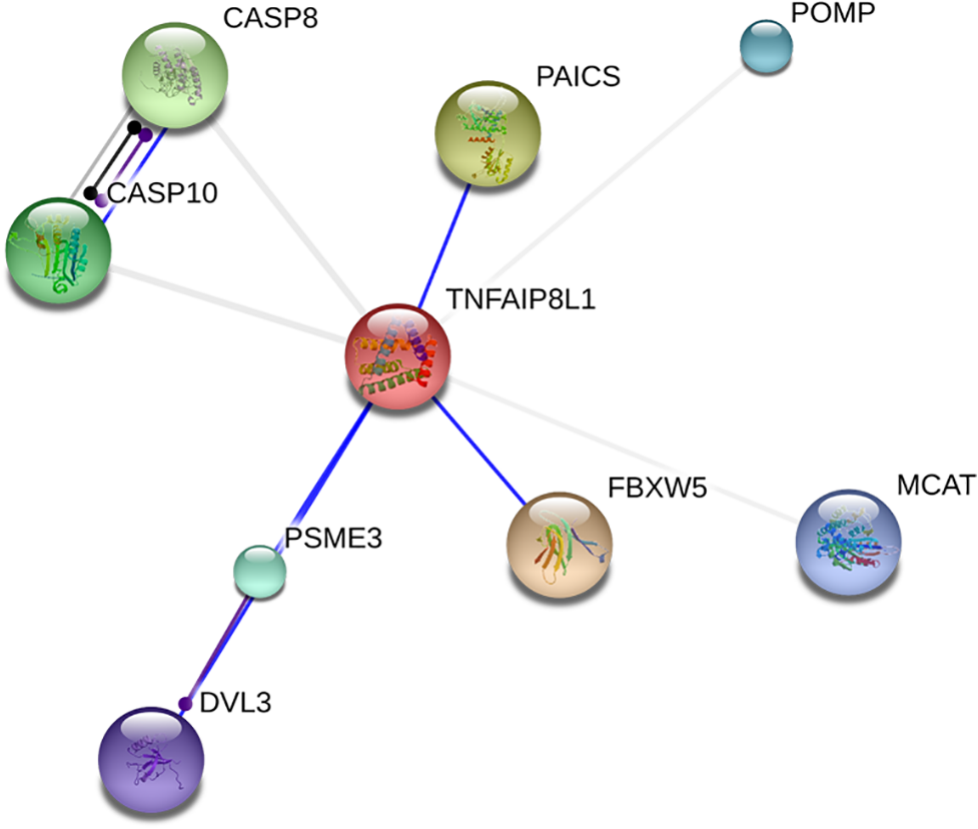
Protein-protein interaction analysis for TIPE1. STRING platform is used to predict protein interactions. FBXW5, caspase8, caspase10, POMP and et al. were predicted to be interacted with TIPE1.

STRING platform is a database of known and predicted protein interactions [11,12]. As the results shown in Fig 6, TIPE1 protein might be interacted with F-box and WD repeat domain containing 5 (FBXW5), caspase8, caspase10, proteasome maturation protein (POMP), et al. The maximum score is 0.812 from FBXW5 protein.

## Discussion

Recently, the expression of TNFAIP8 family has been reported to be altered in the development of many types of cancers, including HCC, intestinal-type gastric adenocarcinoma, lung cancer, epithelial ovarian cancer, endometrial cancer and colon cancer [13–18]. TNFAIP8 family has also been demonstrated to be associated with diabetic nephropathy, Parkinson's disease and colitis [5,19,20]. Of all the members of TNFAIP8 family, TIPE2 contains a hydrophobic cavity in the central and it may be response for the critical function of TIPE2 in immune homeostasis [7]. TIPE1 is another member of TNFAIP8 family and it exhibits a high degree of sequence homology with TIPE2 [7]. Although a common fold exists both in TIPE2 and TIPE1, TIPE1 may not play an essential role in immunity [21]. Cui et al. [21] have reported that TIPE1 was not detected in mature T and B lymphocytes. Nevertheless, it was expressed in a human B lymphoblast cell line HMy2.CIR, which is transformed with Eptein-Barr Viral DNA [21]. In addition, TIPE1 was also expressed in various tissues of mice, including brain, liver, kidney, stomach and so on, and it was up-regulated in various carcinoma cell lines, especially the cell lines transformed with virus [21]. However, Zhang et al. [9] have also reported that TIPE1 expression was down-regulated in HCC tissues, compared with that in adjacent non-tumor tissues. TIPE1 could induce apoptosis and inhibit cell growth of HCC cells [9]. Hence, the interesting and different expression levels of TIPE1 in various cells attract our attention to its structure.

In this study, we attempted to analyze its structure using the bioinformatic method in web platform. Since the sequences of TIPE1 may be well conserved in mammals, the sequence of human TIPE1 was then chosen to further analyze the structure of TIPE1 and SignaIP platform was performed to predict the signal peptide of TIPE1, while TMpred server was used to predict the transmembrane region of TIPE1. According to the arithmetic of SignaIP platform and TMpred system, no signal peptide and no transmembrane domain was speculated in human TIPE1. In addition, ProtParam was also performed to compute various chemical and physical parameters and the results showed that the aliphatic index of TIPE1 was 100.75, which represented a positive factor for the increase of the thermo stability of a globular protein. Meanwhile, the results of prediction for post-translational modifications of TIPE1 showed that four kinds of phosphorylation sites were speculated to be in TIPE1 protein, while PKC site and PKA site may be also existed in TIPE1 protein. We also constructed the 3D structure of TIPE1 and the results also showed that the predicted structure was stable.

Importantly, TIPE1 was predicted to interact with some proteins, including FBXW5, caspase8 and caspase10 proteins. As the results of protein-protein interaction analysis for TIPE1, TIPE1 may induce apoptosis or autophagy through these molecules in some cells. Previous study of TIPE1 function on hepatocellular carcinoma (HCC) cells demonstrated that TIPE1 could induce apoptosis of HCC cells by negatively regulate the expression of Rac1 [9]. Ha et al. also found that TNFAIP8 l1/Oxi-β could increase autophagy in Parkinson’s disease model through binding with FBXW5 [19]. These studies may support our view. Interestingly, another member of TNFAIP8 family, TIPE2, was also reported to inhibit the expression of Rac1 to suppress metastasis and invasion of HCC cells [22]. In addition, caspase8 was also reported to bind to TIPE2 protein to induce cell apoptosis [2]. TIPE2 can promote apoptosis of lung cancer cells trough caspase3 and caspase9 [23]. These studies all suggest that the function of TIPE1 on cell apoptosis and autophagy needs to be focused on in the further study.

In conclusion, TIPE1 may be a stable protein with no signal peptide and no transmembrane domain and TIPE1 might induce apoptosis and autophagy in some appropriate cells. All the results of bioinformatic analysis of TIPE1 will provide the basis for the further study on the function of TIPE1 on some diseases.
